# Exceptional Antibodies Produced by Successive Immunizations

**DOI:** 10.1371/journal.pbio.1002321

**Published:** 2015-12-07

**Authors:** Patricia J. Gearhart, Diana P. Castiblanco, Lisa M. Russell Knode

**Affiliations:** Laboratory of Molecular Biology and Immunology, National Institute on Aging, National Institutes of Health, Baltimore, Maryland, United States of America

## Abstract

Antibodies stand between us and pathogens. Viruses mutate quickly to avoid detection, and antibodies mutate at similar rates to hunt them down. This death spiral is fueled by specialized proteins and error-prone polymerases that change DNA sequences. Here, we explore how B lymphocytes stay in the race by expressing activation-induced deaminase, which unleashes a tsunami of mutations in the immunoglobulin loci. This produces random DNA substitutions, followed by selection for the highest affinity antibodies. We may be able to manipulate the process to produce better antibodies by expanding the repertoire of specific B cells through successive vaccinations.

## Abstract 2

This Essay explores the possibility of manipulating somatic hypermutation in B lymphocytes by administering serial vaccinations with engineered antigens, with the aim of generating potent antibodies to destroy pathogens.

## Mutated Antibodies

*The War of the Worlds*, written by H. G. Wells and published in 1898, tells the story of invading Martians who covet our earth. They lay waste to the landscape by destroying everything with their heat rays and tripod machines. Civilization is doomed. Suddenly, the Martians fall dead, brought down by microbial infections, because they had no immunity to earth’s bacteria. This scenario would not happen to most humans because they could respond to Martian bacteria with somatically mutated antibodies. The Martians may have lacked the protein, activation-induced deaminase (AID), which initiates DNA mutations. However, we are capable of recognizing foreign antigens that the human race has never seen before and has no immunity to. B cells are first activated by binding to these antigens with low affinity and then expressing AID to introduce random mutations into antibody genes. The B cells that, by chance, express high affinity immunoglobulin receptors are selected, expanded, and differentiate to produce large amounts of secreted antibody to hunt down the alien invaders.

In fact, B cells make drugs, that is, antibodies, and it is worth knowing how these drugs are created. In this Essay, we explore what triggers an ordinary antibody to become an elite player. As proof of concept, the frontline defense against novel, exotic diseases such as Ebola is to administer antibodies from people who have survived [1]. The characteristics of exceptional antibodies suggest that immunization should proceed with a successive series of antigens. First, the repertoire of B cells could be expanded with less specific antigens to generate many different B cells bearing low affinity receptors with a few mutations. Second, rare crossreactive cells in this repertoire could be selected with a more restricted antigen in order to induce them to further mutate to produce high affinity antibodies.

## How Somatic Hypermutation Works

**Fig 1:**
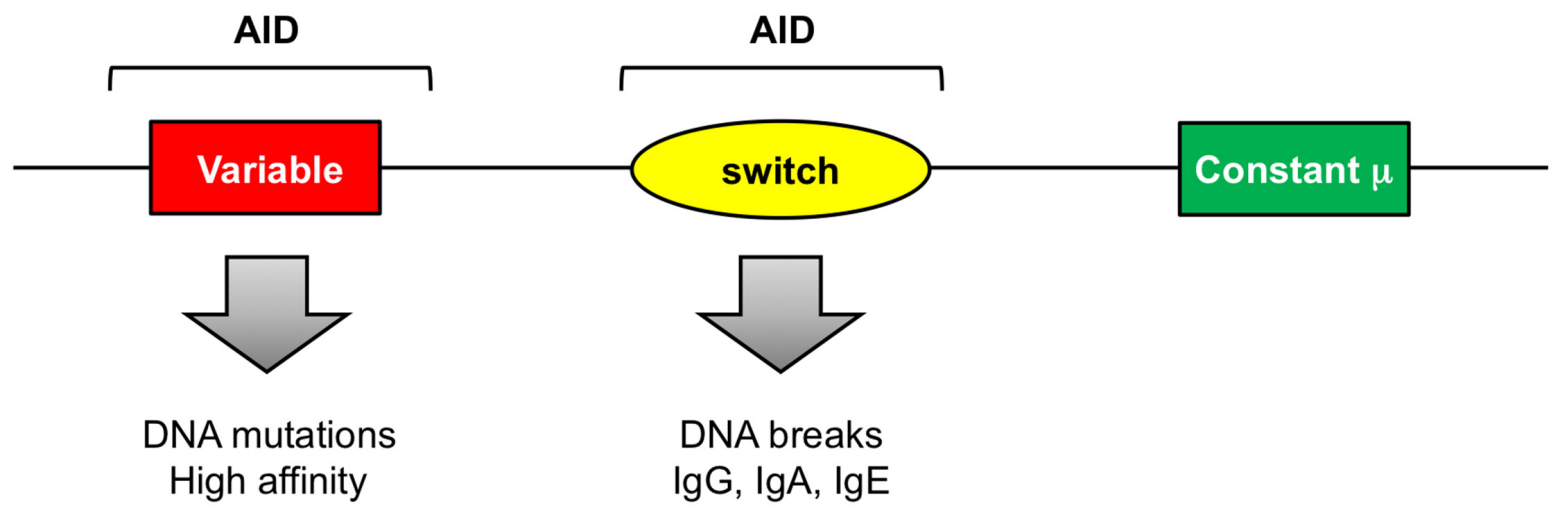
AID is directed to variable and switch regions on the immunoglobulin loci. Mutations in variable genes change amino acids to encode high affinity antibodies. Mutations in the switch regions preceding constant genes on the heavy chain locus cause double-strand breaks to recombine from the initial IgM to IgG, IgA, and IgE antibodies.

AID is only expressed in activated B cells and is specific for the immunoglobulin loci encoding heavy chains and kappa and lambda light chains [2,3]. The protein is then targeted to variable genes and switch regions through a poorly understood mechanism (Fig 1). Recently, transcription has been suggested to shepherd AID to these regions [4–11]. AID-induced mutations include both nucleotide substitutions for changing variable gene codons, and DNA strand breaks for switching from IgM to IgG, IgA, and IgE. These two steps, mutation and switching, define an antibody’s purpose: (a) to bind to an epitope on a pathogen with specificity and strength, and (b) to eliminate the pathogen via the heavy chain’s interaction with complement and phagocytes. As part of the diabolical twist in this mutation-generating scenario in B cells, the pathway appears to hijack some of the proteins involved in canonical DNA repair. This is indeed one of the most amazing aspects of the mutation machinery. Proteins are abducted from two repair pathways that were recently spotlighted in the 2015 Nobel Prize awards in Chemistry: base excision repair and mismatch repair. Another surprising aspect is the extensive use of error-prone DNA polymerases to introduce nucleotide base changes. Thus, somatic hypermutation uses an unusual DNA deaminase, a handful of DNA repair proteins, and several low fidelity polymerases to generate extensive mutations and breaks in B cells [12].

## Characteristics of Potent Antibodies

An effective antibody will bind to an antigen with high affinity, or strength of binding, which allows the antibody to bind lower concentrations of antigen. High affinity can be generated by somatic changes in the variable genes. What does somatic hypermutation actually look like following immunization? The variable region encoded by the variable gene segment is divided into three framework regions (FWR) and two complementarity determining regions (CDR). FWRs, which form the basic structure of the protein, generally have fewer mutations than CDRs, which contact the antigen and are thus selected for high affinity. For example, antibodies to influenza virus hemagglutinin contain about ten mutations per heavy chain variable gene segment, and certain amino acids are repeatedly found in CDRs, which contact the virus [13].

**Fig 2:**
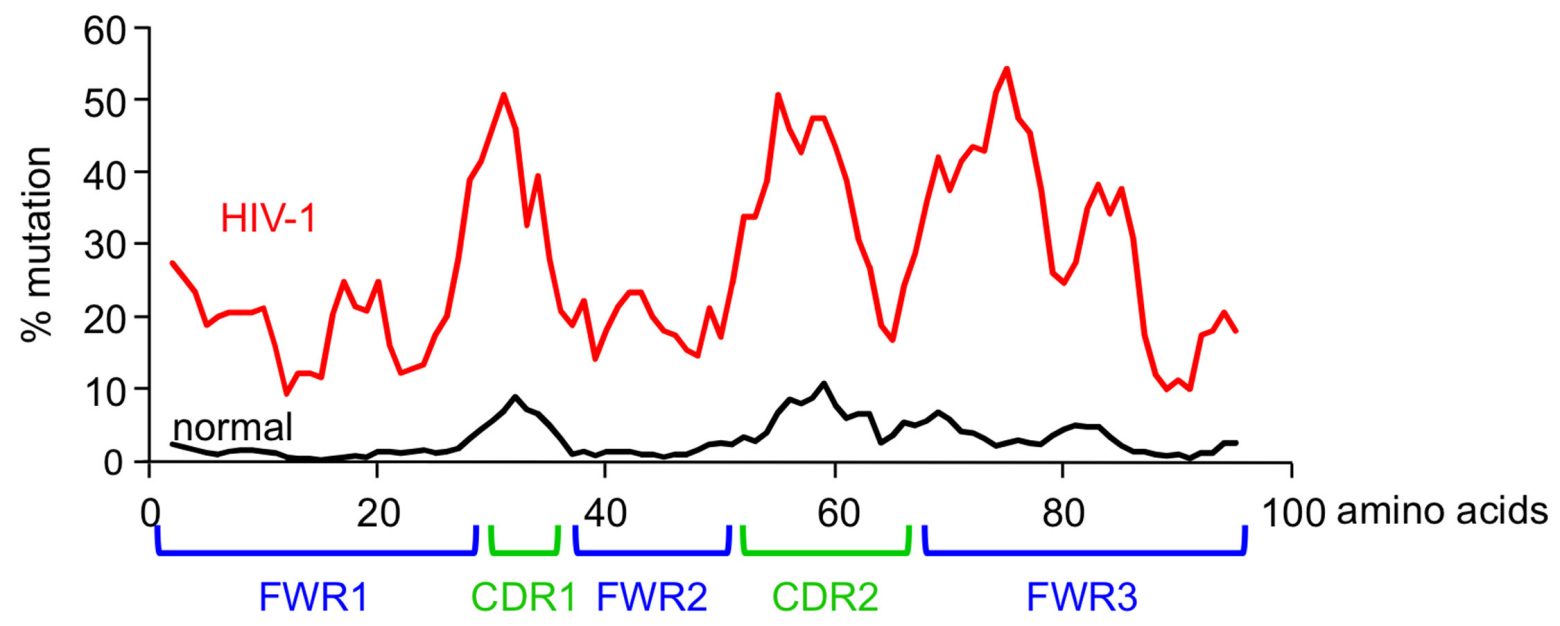
Frequency of nucleotide mutations across the heavy chain variable gene segment in normal (black) and HIV-1 (red) antibodies. The *x*-axis shows amino acid numbering of FWR and CDR areas. Data adapted from Rosner et al. [22] and Scheid et al. [21].

Another cadre of antibodies that has been extensively sequenced to examine their pattern of mutation is broadly neutralizing antibodies to HIV-1. During the last 30 years, antibodies to HIV-1 were originally thought to be ineffective because they were unable to elicit protective responses. However, recent groundbreaking work by a number of labs has uncovered a group of elite antibodies made by patients who survived the disease [14–17]. These antibodies take approximately 2–4 years to arise in humans, and they bind to the CD4 binding site on the virus, which blocks the virus from entering T cells [18–20]. The antibodies are potent and broad because they have strong neutralizing activity to a wide spectrum of HIV viruses. They are encoded by a handful of variable genes for the heavy and light chains. The striking observation made by Scheid et al. [21] was that the antibodies are riddled with mutations, at a 10-fold higher frequency than observed in normal antibodies [22], or about 80 mutations per heavy chain variable gene segment. Not only are one-third of the nucleotides mutated, an astounding number, but the mutations occurred in FWRs as well as CDRs. As shown in Fig 2, the frequency of mutations in FWR3 was as high as that in CDR2. What does this mean? The FWRs must contact HIV-1 as well as the CDRs, and those mutations are selected for binding [23]. In fact, when the antibodies are experimentally reverted back to their nonmutated, germline sequence, they do not bind HIV-1 [21,24–27]. It is estimated that the rate of antibody mutation equals that of HIV-1 mutations [28–30], confirming the ongoing arms race between B cells and viruses. Furthermore, these broadly neutralizing antibodies have the potential for use in antibody-based vaccines to treat HIV-1 [31]. In contrast to active immunization, which occurs by injecting the antigen, passive immunization occurs by injecting the antibody. These exceptional antibodies have been shown to be protective and reduce viral loads in animal models, suggesting their therapeutic potential in humans [32,33]. Indeed, stunning results have been recently observed after administering a single, broadly neutralizing antibody to HIV-1-infected humans, which significantly reduced the viral load [34]. Protection by passive immunization occurs immediately and can last for months, whereas active immunization has the potential to last for years. Thus, the best long-term scenario would be to induce immunity with antigen, which could engage all the weapons in the immune arsenal, including T cells and macrophages.

## Two Layers of Immunity

It takes time after immunization with antigen to generate antibodies with a high frequency of mutations, particularly in the FWRs. Current vaccines are focused on containing conserved epitopes that are shared among subtypes of pathogens, such as the CD4 binding site of HIV-1 or the stem of influenza hemagglutinin [35]. More recent strategies for HIV-1 are to immunize mice with antigens that bind to the germline antibodies to induce a first wave of mutation and then to boost with a specific viral antigen to generate a second wave of mutation [36–39]. However, this approach requires knowing what the germline antibody is, which may be unknown in some diseases. Furthermore, individuals may not inherit or express a given variable gene, and exposure to different environments could alter the rate of mutation. We propose a more general strategy to induce a primary layer of immunity before infection using a plethora of engineered antigens to develop a large repertoire of primed B cells that could last for years. This would supply a diverse set of variable genes with a few mutations, preferably in FWRs as well as CDRs, and generate B cells with low affinity for the pathogen. In this shotgun approach, the primed memory cells could then be activated by a hair trigger, such as a much lower dose of a specific antigen. The boosting antigen could be chosen at will to represent the latest incarnation of a rapidly mutating virus or bacteria. This secondary layer of immunization would select rare crossreactive or heteroclitic B cells from the expanded repertoire. Contrary to dogma that antibodies have exquisite specificity for the original, primary antigen, heteroclitic antibodies are known to bind strongly to antigens with little or no similarity [40–42]. These astonishing antibodies could then continue to undergo mutation in germinal centers and generate high affinity proteins with specificity for the secondary antigen.

However, there is collateral damage in any immunization protocol, whether it is during the primary or secondary immunization. One downside is the potential generation of autoimmune antibodies through mutation or crossreaction [43]. Autoreactivity could be monitored by testing serum antibodies for autoreactivity after the primary immunization, and the vaccine composition could be adjusted accordingly. Another problem is mistargeting of the mutator protein, AID. Thus, in addition to generating extreme diversity in the immunoglobulin loci, AID can direct a potential suicidal path for B cells. Their DNA is blanketed with mutations and breaks, which sometimes escape the immunoglobulin loci to end up in other genes, provoking lymphomagenesis [44,45]. This is certainly a very rare event, as the vast majority of immunizations do not produce cancer or even autoimmunity. In conclusion, the two-layer approach of a broad immunization with a group of related antigens, perhaps representing previous subtypes of a pathogen, may expand the repertoire of memory B cells. Such a cocktail of engineered antigens could preprime people to quickly respond with exceptional antibodies, even to Martian bacteria.

## Abbreviations

AID: activation-induced deaminase
CDR: complementarity determining region
FWR: framework region
